# Effects of intercalated atoms on electronic structure of graphene nanoribbon/hexagonal boron nitride stacked layer

**DOI:** 10.1038/s41598-019-39719-9

**Published:** 2019-03-06

**Authors:** Dongchul Sung, Gunn Kim, Suklyun Hong

**Affiliations:** Department of Physics & Astronomy and Graphene Research Institute, Sejong University, Seoul, 05006 Republic of Korea

## Abstract

Using first-principles calculations, we investigate an atomic impurity at the interface of a van der Waals heterostructure (vdW heterostructure) consisting of a zigzag graphene nanoribbon (ZGNR) and a hexagonal boron nitride (h-BN) sheet. To find effects of atomic intercalation on geometrical and electronic properties of the ZGNR on the h-BN sheet, various types of impurity atoms are considered. The embedded atoms are initially placed at the edge or the middle of the ZGNR located on the h-BN sheet. Our results demonstrate that most of the impurity atoms are more stable at the edge than at the middle in all cases we consider. Especially, a nickel atom has the smallest energy difference (~0.15 eV) between the two embedding positions, which means that the Ni atom is relatively easy to intercalate in the structure. Finally, we discuss magnetic properties for the vdW heterostructure with an intercalated atom.

## Introduction

Graphene has attracted tremendous attention as a next-generation electronic material because of its unique chemical and physical properties^1–4^. Mica^5^, silicon carbide (SiC)^6,7^ and silicon dioxide (SiO_2_)^1,8–11^ have been usually used as substrates for nanodevices. However, such types of substrates reduce the electronic mobility of graphene in the device^12,13^. Recently, the concern with the hexagonal boron nitride (h-BN) sheet as an insulating substrate for the graphene devices has been growing. When graphene is transferred directly to the conventional substrates such as SiO_2_, the electrical mobility is decreased by charged impurities in the substrates. Besides, the surface roughness of the SiO_2_ substrate results in the large corrugation of graphene^12,14–16^. In contrast, graphene on the h-BN sheet is almost flat, and shows much higher electrical mobility than graphene on SiO_2_. The h-BN sheet is an insulator with a large optical bandgap of about 6 eV^17^ and small lattice mismatch with graphene about 1.6%. Another important point is that h-BN has weak van der Waals (vdW) interaction with graphene. Therefore, h-BN may be a good candidate as a dielectric substrate for graphene-based nanodevices. Besides, this material is chemically inert, compared to metal surfaces.

The free-standing zigzag graphene nanoribbon (ZGNR) shows localized electronic states and antiferromagnetic property at the edge^18–20^. The edge states of the ZGNR can be affected by impurity atoms or the supporting substrate. Using first-principles calculations, Choi *et al*. studied the adsorption properties of alkali metal atoms from the edge to the middle of ZGNRs^21^. The adsorption energies of alkali metal atoms on the ZGNR depend on the position of the impurity, and are largest at the ZGNR edge. On the other hand, Lee *et al*. considered the functionalization of halogen atoms and molecules at the ZGNR edge^22^. They focused on free-standing ZGNR for the adsorption of atomic impurities. Since the real nanodevices are fabricated on a substrate, however, we need to study intercalation properties of such defect atoms at interfaces of graphene/substrate systems.

In this paper, we report a first-principles study of atomic and electronic properties of a ZGNR on an h-BN sheet with an intercalated atom. The purpose of our work is to understand intercalation properties of extrinsic defects such as alkali metal (Li, Na, and K) and halogen (Cl, Br, and I) atoms in order to tailor the localized states of the zigzag edge in a graphene on an h-BN sheet. We also examine effects of other types of residual atoms (Cu, Ni, and Si atoms) between graphene and h-BN sheet, which could be adsorbed to the graphene or h-BN sheet in the transfer process. The intercalated atoms are expected to bring about charge doping or to change the magnetic properties of the system.

## Results and Discussion

### Van der Waals ZGNR/h-BN heterostructures

**Figure 1:**
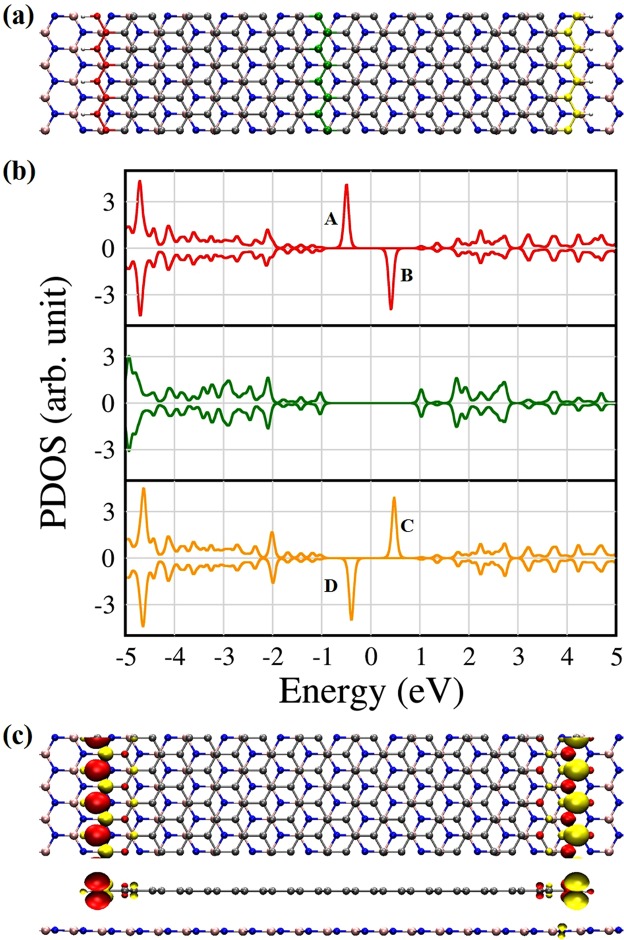
(**a**) Top view of a vdW ZGNR/h-BN heterostructure, (**b**) its PDOS, and (**c**) top and side views of spin densities of the model system. Each coloured line shown at the ZGNRs corresponds to the same coloured line of the PDOS. The red, green, and yellow colours represent the 1st, 9th, and 18th lines from the left ZGNR edge, respectively. A, B and C, D represent the spin-up and down states at the left and right edges, respectively.

First, we obtain the optimized geometry of the vdW ZGNR/h-BN heterostructure with no intercalated atom, as shown in Fig. 1(a), where the grey, blue, and pink colours represent carbon, nitrogen, and boron atoms, respectively. The most stable configuration resembles the Bernal stacking of graphite. The carbon atoms of the ZGNR are located on top of the boron atoms and the centre of h-BN hexagons; that is, the centres of the ZGNR hexagons are on top of the nitrogen atoms of h-BN. Our results are in consistent with the previous reports^20,23–26^. The equilibrium distance between the ZGNR and the h-BN sheet is 3.15 Å. We find that the localized states exist at the edges of the ZGNR on the h-BN sheet, represented by the almost flat band of the zigzag edge around the Fermi level (E_F_). The localized states^27^, however, rapidly decay into the bulk, as shown in Fig. 1(b). Figure 1(b) shows the projected densities of states (PDOSs) at the left and right edges and near the middle of the ZGNR, respectively. The red, green, and yellow lines of the PDOSs of the ZGNR correspond to the same colours of carbon atoms at the ZGNR in Fig. 1(a), respectively. The results are same as those for free-standing ZGNRs^28^. The PDOS plots imply that the h-BN sheet does not affect the electronic properties of the ZGNR, in contrast to the conventional substrates such as SiC and SiO_2_. We also calculate spin densities to examine the magnetic properties of the edge states of the ZGNR. As in the ZGNR without a substrate, antiferromagnetic properties are clearly shown in the ZGNR on the h-BN sheet, which means that spin-up and spin-down electrons are localized at each edge of the ZGNR, as shown in Fig. 1(c).

For the ZGNR placed on the h-BN sheet, we assume that the ZGNR edges are terminated by hydrogen atoms. The space between the H-terminated edge of the ZGNR and the h-BN sheet may open rather wide, and the open-edged ZGNR on h-BN may allow atoms or molecules to be intercalated between the ZGNR and the h-BN sheet. We place the alkali (Li, Na, and K), halogen (Cl, Br, and I), and other impurity (Cu, Ni, and Si) atoms at both edge and deep positions. We find the stable positions for the intercalated atoms located at edge and deep positions. The binding energy of each impurity atom between the ZGNR and the h-BN sheet is defined as

$${{\rm{E}}}_{{\rm{b}}}={{\rm{E}}}_{{\rm{tot}}}({\rm{impurity}})+{{\rm{E}}}_{{\rm{tot}}}({\rm{ZGNR}}/{\rm{h}} \mbox{-} \mathrm{BN})-{{\rm{E}}}_{{\rm{tot}}}(\mathrm{ZGNR}/\mathrm{impurity}/{\rm{h}} \mbox{-} \mathrm{BN}),$$

where E_tot_ represents the total energy of the optimized geometry  for each system. Here, the positive (negative) sign of the binding energy represents an exothermic (endothermic) process. The binding energy values are listed in Table 1. Our results show that the deep position is less stable than the edge position because of the large curvature and strain resulted from the deformation of the graphene and h-BN sheet. For all the halogen atoms we consider, the deep position has negative binding energies, and is energetically unstable. The Li atom is small, and may be incorporated at the deep position since the difference of the binding energy is only ~0.21 eV between the deep position (E_b_ = 2.40 eV) and the edge position (E_b_ = 2.61 eV). One could produce a new type of vdW nanomaterials using the Li doping because the Li impurity atom tends to donate electron to the host material, and change the electronic structure of the system. The energy difference of the Ni atom for edge and deep positions is the smallest among them (~0.15 eV), whereas that for the halogen family is very large owing to a strong chemical bond to the edge of the ZGNR. For comparison with the results, we calculate the binding energies of the impurity atoms on the top surface of the ZGNR and the bottom surface of the h-BN sheet in the vdW ZGNR/h-BN heterostructure. The calculational results are summarized in Tables S1 and S2 (see Supplementary Information (SI)).

**Table 1:** Binding energy (in eV) of the intercalated atoms between the ZGNR and the h-BN sheet.

Alkali			Halogen			Others		
	Edge	Deep		Edge	Deep		Edge	Deep
Li	2.61	2.40	Cl	2.09	−1.10	Cu	1.88	1.33
Na	1.46	1.04	Br	1.63	−1.59	Ni	3.54	3.39
K	1.24	0.40	I	1.18	−2.09	Si	2.09	−0.50

### Van der Waals ZGNR/h-BN heterostructures with alkali metal and halogen atoms

**Figure 2:**
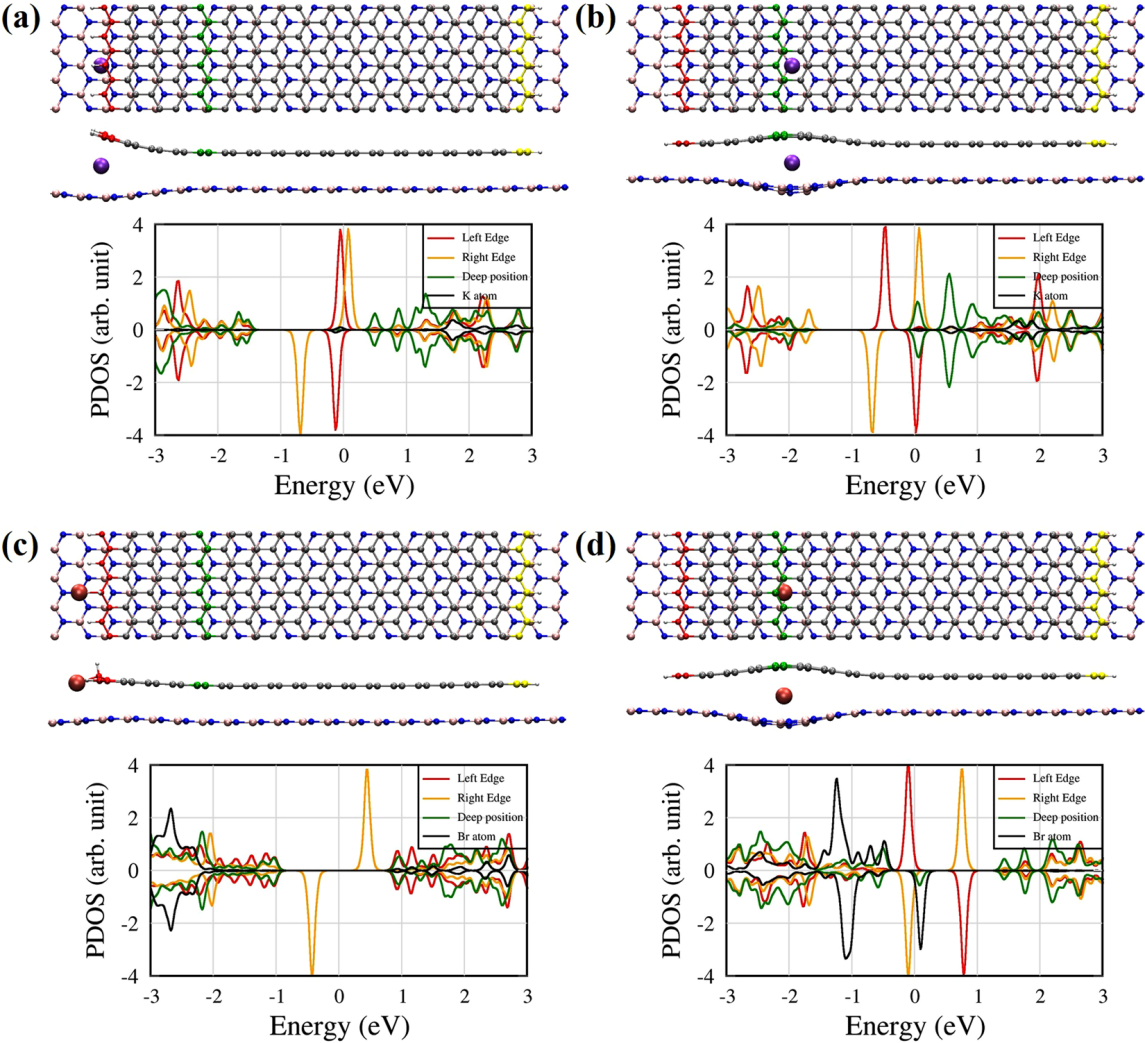
Atomic and electronic structures of vdW ZGNR/h-BN heterostructures  with intercalated (**a**,**b**) potassium and (**c**,**d**) bromine atoms at the edge and deep positions, respectively. Each colour line shown at the ZGNRs correspond to the same colour solid line of the PDOS. The red, green, and yellow atoms at the ZGNR represent the 1st, 5th, and 18th line from the left ZGNR edge, respectively.

To investigate effects of the intercalated atoms at the interface of the ZGNR/h-BN structure, we calculate the atomic and electronic structures of alkali metal and halogen atoms placed at both edge and deep positions, as shown in Fig. 2. Similar trends in atomic and electronic structures are shown for alkali metal and halogen atoms. We choose the K and Br atoms for a detailed study. Figure 2 shows top and side views of optimized geometries and the PDOSs of the ZGNR/impurity/h-BN structures for K and Br impurities. Obviously, the interstitial space between the ZGNR and the h-BN sheet is enlarged in the presence of the intercalated atom at both edge and deep positions, as shown in Fig. 2. As shown in Fig. 2(a,b), neither h-BN nor ZGNR makes a chemical bond with the K atom for both edge and deep positions. The red, yellow, and green lines of the PDOS correspond to the left edge, right edge, and deep positions of ZGNRs, respectively, and the black line of the PDOS represents the impurity atom. Electron transfer occurs from the K atom to the ZGNR and the h-BN sheet at both edge and deep positions. At the edge position, the K atom has weak orbital hybridization with the ZGNR around E_F_, as shown in Fig. 2(a). Here, we find the broad potassium bands in the conduction band (~1.8 eV). It means that the K atom is fully ionized, and donates an electron to the ZGNR. Interestingly, the left edge near the K impurity does not have any practical spin-polarized state, whereas the right edge still has a spin-polarized state like a defect-free ZGNR edge. For both edge and deep positions, the strong edge state of the ZGNR occurs at E_F_, and the trend of electron donation is expected to be very similar. Therefore, one could use the partially filled edge states as one-dimensional channels for quantum electronic transport.

In contrast to the potassium impurity, the bromine atom forms a chemical bond to a carbon atom of the left edge of the ZGNR with sp3-like hybridization, and the left edge states of the ZGNR disappear in the edge-position configuration. Because of strong electron affinity of Br, the ZGNR is hole-doped, and the PDOS of the Br atom is clearly shown between −3.0 and −2.0 eV. More interestingly, the Br atom at the deep position creates spin magnetic moments coupled to the ZGNR edge, which will be discussed below in more detail, although it forms no chemical bonds to the ZGNR or the h-BN sheet. At the deep position, the narrow DOS peaks of Br occur near E_F_ for down-spins, and the localized edge states of the ZGNR are shown near E_F_ in Fig. 2(d). Thus, the Br-originated localized state near E_F_ can cause electron scattering.

### Van der Waals ZGNR/h-BN heterostructures with other types of atoms

**Figure 3:**
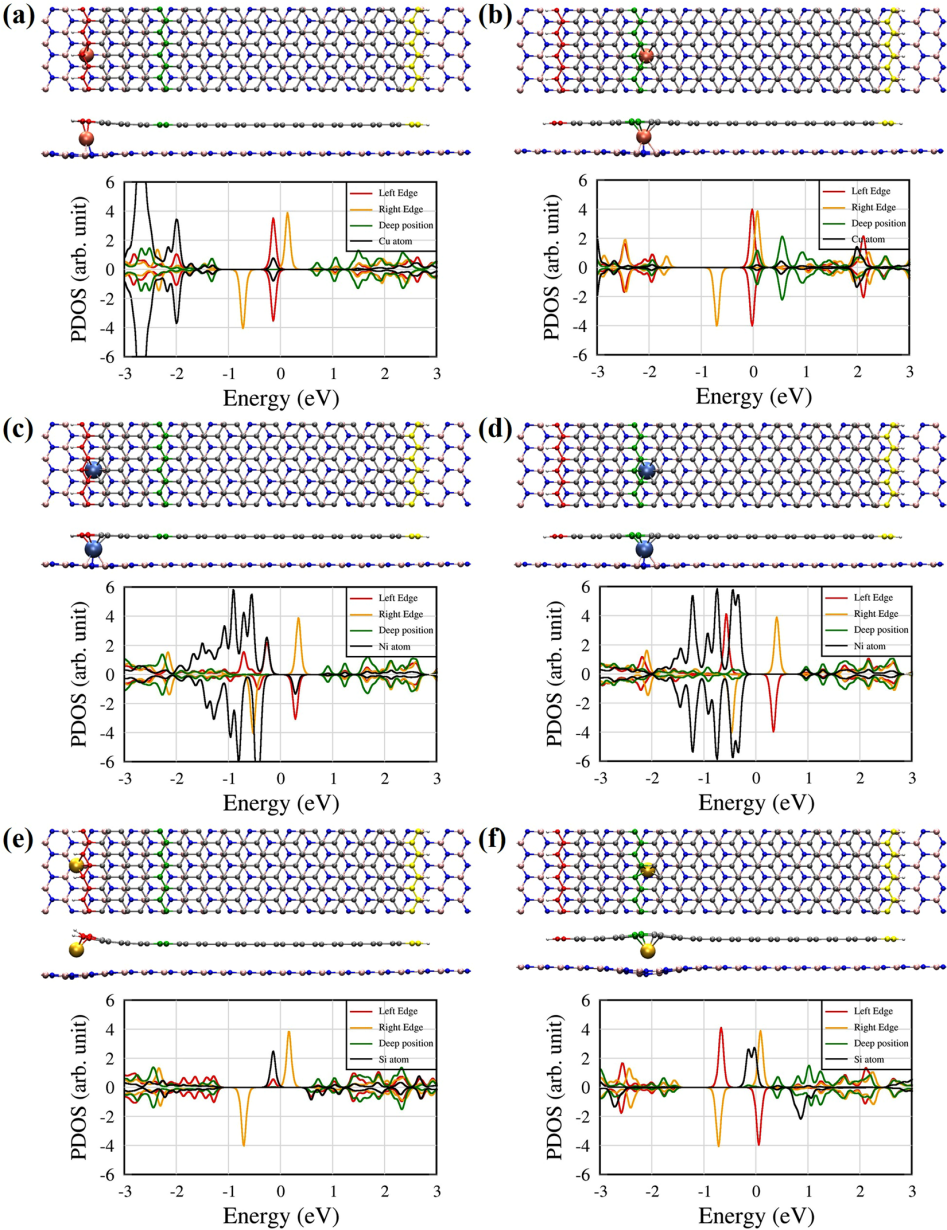
Atomic and electronic structure of the ZGNRs on the h-BN sheet with intercalated (**a**,**b**) Cu, (**c**,**d**) Ni, and (**e**,**f**) Si atoms at the edge and deep positions, respectively. The carbon atoms of red, green, and yellow colours at the ZGNRs correspond to the same colour solid lines of PDOS. The red, green, and yellow atoms at the ZGNRs represent the 1st, 5th, and 18th line from the left ZGNR edge, respectively.

As mentioned above, in the transfer process, some residual atoms from a substrate such as Cu, Ni, and SiC may be adsorbed on the h-BN or graphene surface^29^. In such cases, the deep-position configuration of the graphene/impurity/h-BN structure may be formed. Therefore, we also consider the deep-position configuration as well as the edge-position configuration for Cu, Ni and Si defects in order to check the impurity effect on the electronic properties and spin magnetic properties of the ZGNR, although the edge configuration is energetically more stable than the deep-position configuration. Figure 3 shows the atomic and electronic structures of the vdW ZGNR/h-BN heterostructures with Cu, Ni, and Si defects at edge and deep positions, respectively. The Si atom results in significant deformation of the ZGNR and the h-BN sheet, but the Cu and Ni atoms do not cause remarkable geometrical changes in the two layers at the deep positions. The large and narrow PDOSs originating from Cu 3d orbitals are shown around −3 eV, and the Cu 4 s electron is slightly hybridized with the ZGNR edge states at E_F_. For both the edge and deep positions, electron transfer takes place from the Cu atom to the ZGNR, and cancels the spin magnetic moment of the left edge of the ZGNR, as shown in Fig. 3(a,b). For the Ni impurity, the Ni 3d orbitals are hybridized with the localized C 2p orbitals at the ZGNR edge between −2.0 and 0 eV (E_F_), as shown in Fig. 3(c,d). Figure 3(e) shows that the Si impurity atom bonds to the ZGNR edge and makes DOS peak just below E_F_. On the other hand, the Si atom at the deep position produces two nearly-degenerate localized states just below E_F_, as shown in Fig. 3(f).

### Magnetic properties of van der Waals ZGNR/h-BN heterostructures with impurity atoms

**Figure 4:**
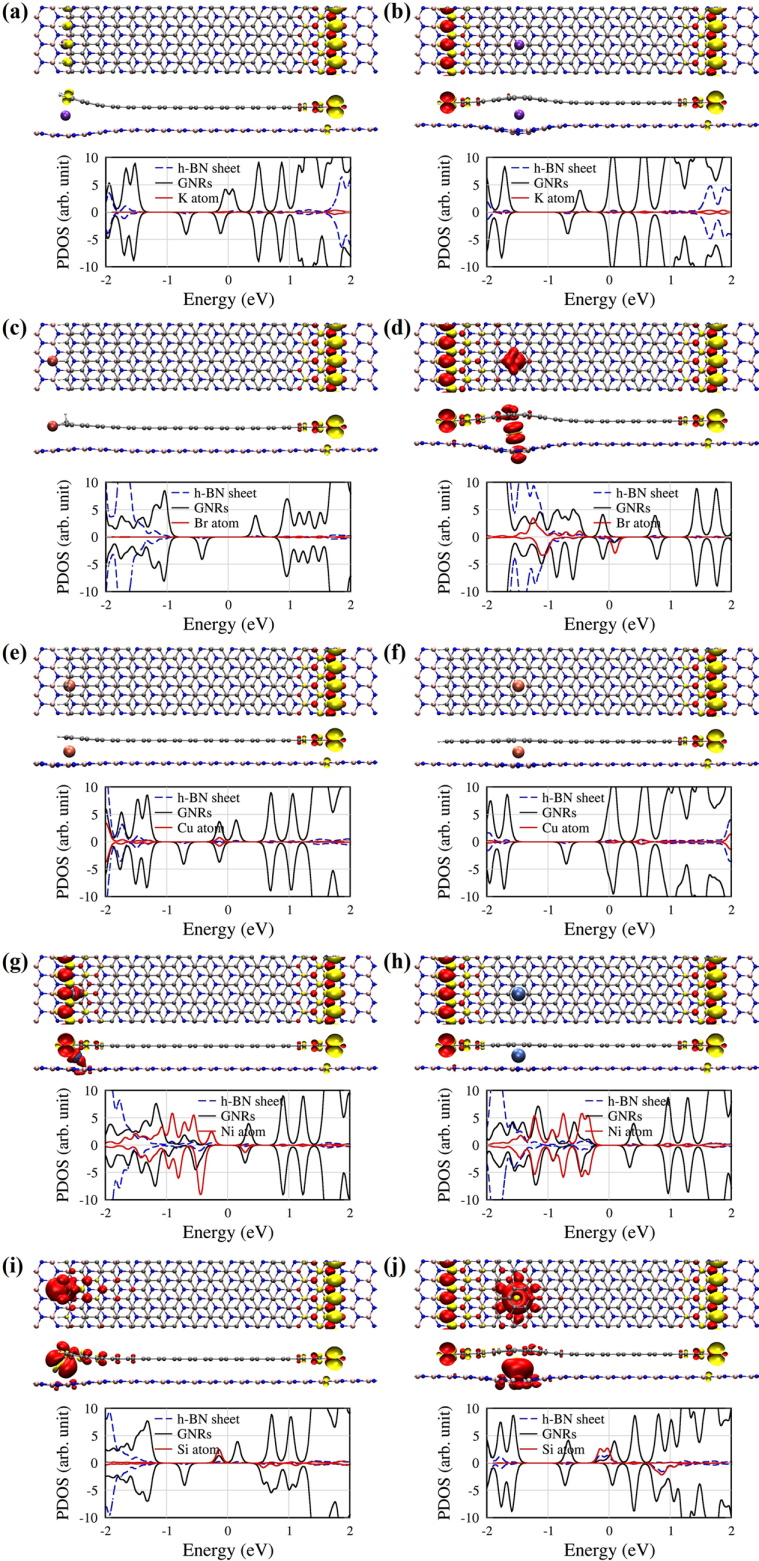
Spin densities and the PDOS for (**a**,**b**) K, (**c**,**d**) Br, (**e**,**f**) Cu, (**g**,**h**) Ni, and (**i**,**j**) Si atoms at the edge and deep positions, respectively. The red and yellow balloons represent he spin-up and spin-down states, respectively. The black, red, and blue dotted lines in the PDOS represent the ZGNR, impurity, and h-BN sheet, respectively.

Finally, we explore spin magnetic properties of the ZGNR/impurity/h-BN structures. Figure 4 shows the spin densities and the PDOS for ZGNR/h-BN structures with intercalated atoms (K, Br, Cu, Ni and Si) at both edge and deep positions. The red and yellow colours represent the spin-up and spin-down electrons, respectively. Table 2 summarizes the spin magnetic moment of each system. ZGNRs have localized states at the edge, and have the opposite spin configuration between two edges^19^. For alkali metal atoms at the edge position, the net spin magnetic moment of the system is ~1.0 μ_B_ (the Bohr magneton, 1 μ_B_ ≈ 9.274 × 10^−24^ J T^−1^). For the edge position, it is interesting that electron transfer takes place from the incorporated alkali metal atom to a ZGNR edge so that the spin magnetic moment of the zigzag edge bonded to the alkali metal atom disappears; however, the spin configuration of the opposite edge retains, as shown in Fig. 4(a). For the deep position, the antiferromagnetic configuration in the ZGNR is almost kept [see Fig. 4(b)], but electrons donated from the alkali metal atom are redistributed to give magnetic moment of 0.2 μ_B_.

**Table 2:** Net spin magnetic moments (in μ_B_) of the ZGNR/impurity/h-BN structures at edge and deep positions.

Alkali			Halogen			Others		
	Edge	Deep		Edge	Deep		Edge	Deep
Li	0.94	0.13	Cl	1.00	0.92	Cu	0.98	0.92
Na	0.98	0.16	Br	1.00	0.96	Ni	0.00	0.00
K	0.98	0.21	I	1.00	0.99	Si	0.00	1.63

For the edge position, on the other hand, the halogen atom forms a chemical bond to an edge of the ZGNR, and the spin magnetic moment results only from the opposite site of the ZGNR, which gives the spin magnetic moment of 1.0 μ_B_. Compared with Fig. 1(b), Fig. 4(c) shows that the localized states at the left edge (A and B states in the Fig. 1(b)) disappear near E_F_. Very interestingly, although the ZGNR keeps an antiferromagnetic configuration for the deep position, an almost vertical distribution of spin-up electrons around the halogen atom occurs as shown in Fig. 4(d), which gives the spin magnetic moment of ~1.0 μ_B_. For the Cu impurity atom, the net spin magnetic moments at both the edge and deep positions are about 1.0 μ_B_. As shown in Fig. 4(e), for the edge position, the left edge of the ZGNR near the Cu atom cancels the spin magnetic moment, and the remaining spin configuration of the right edge gives a magnetic moment. The PDOS also reveals that a filled spin-up state [state A in Fig. 1(b)] moves upward, while an unfilled spin-down state [state B in Fig. 1 (b)] moves downward. They meet at the same energy so that the spin density at the left edge disappears. An interesting point is that the Cu atom at the deep position also affects the spin magnetic moment of the left ZGNR edge, as shown in Fig. 4(f), although the distance between the Cu atom and the left edge is about 1.0 nm. We thus find a similar trend to the case of the edge position that a spin-down edge state moves downward and a spin-up state moves upward in energy for the left edge. The Ni defect induces no net magnetic moment, as previously reported in literature^30,31^, and the ZGNR retains antiferromagnetic, as shown in Fig. 4(g,h). However, it is evidently found that strong hybridization between the ZGNR and h-BN sheets occurs owing to the Ni 3d electrons. On the other hand, the left edge states of the ZGNR almost disappear when the Si atom is bonded to the left edge of ZGNR. Consequently, the net spin magnetic moment becomes zero. The PDOS in Fig. 4(i) shows strongly hybridized states between the Si atom and ZGNR just below E_F_ for spin-up electrons. The Si atom at the deep position induces the spin magnetic moment of ~1.6 μ_B_, while the ZGNR maintain the antiferromagnetic configuration. We find the difference between the PDOSs of the edge and deep positions. For the deep position, the states A and B in Fig. 1(b) re-appear in Fig. 4(j), compared to the edge position, and the spin-up states of the Si atom around E_F_ are hybridized with the h-BN states as well as the ZGNR states. We find the difference between the PDOSs of the edge and deep positions. For the deep position, the edge states of the ZGNR move downward, and two almost-degenerate Si states appear just below E_F_. Therefore, the net spin magnetic moment of ~1.6 μ_B_ comes mainly from the Si atom, as shown in Figs 3(f) and 4(j).

## Conclusions

We have carried out the first-principles calculations to investigate the electronic and atomic structure of the ZGNRs on the h-BN sheet with intercalated atoms such as alkali, halogen, and other atoms. The localized states of a ZGNR edge on the insulating h-BN sheet were observed to show a rapid decay into the bulk. Intercalated atoms located at edge and deep positions are considered. The deep position is less stable than the edge position because of strong deformation of the ZGNR and h-BN sheet. We also investigate the magnetic properties of the systems. In particular, the spin magnetic moments come from the ZGNR edge for the alkali metal defect, whereas they come from the intercalated atoms for the halogen impurity. The incorporating atoms affect the ZGNR edge in the vdW ZGNR/h-BN heterostructure. Therefore, it is important to identify the modification of the edge states of the ZGNR on the h-BN sheet with intercalated atoms in comparison with the free-standing ZGNR and to remove the residual atoms on ZGNRs for the devices.

## Methods

### First-principles calculations

To understand the interaction between the ZGNR and the h-BN sheets with an intercalated atom, we have performed density functional theory (DFT) calculations within generalized gradient approximation (GGA) using the Vienna ab initio simulation package (VASP)^32–34^. The projector augmented wave potentials are employed to describe the potential from the centre of each atom. The energy cut-off for the plane-wave basis is set to 400 eV. Spin polarization is also considered in our calculations. All geometries are optimized until the Hellman-Feynman forces acting on the atoms become smaller than 0.03 eV/Å. To include weak vdW interaction between the adsorbate and the ZGNR, we adopt Grimme’s DFT-D2 vdW corrections based on a semi-empirical GGA-type theory^35^. For the Brillouin-zone interaction, we use a Γ-centered 1 × 3 × 1 grid in the Monkhorst-Pack k-point scheme.

For the study of the doping effect of intercalated atoms, we choose alkali metal (Li, Na, and K) and halogen (Cl, Br, and I) atoms. Alkali metal atoms tend to donate an electron to  graphene, whereas halogen atoms are likely to accept one electron from graphene^36–38^. In addition, we consider Cu, Ni, and Si atoms to investigate the changes caused by the atoms in atomic and electronic structures of the vdW ZGNR/h-BN heterostructure since Cu, Ni, and SiC are often used for growing graphene. If the atoms originating from the substrate surface are not removed perfectly during transfer, the graphene or h-BN sheet may contain the impurity atoms on its surface. To study effects of residual atoms, therefore, we consider the impurities such as Cu, Ni, and Si atoms.

The supercell size is 65.33 × 10.06 × 25 Å^3^. The vdW ZGNR/h-BN heterostructure consists of a stacked structure of a single layered h-BN sheet and the *N* = 18 ZGNR. The width of the ZGNR is about 38 Å. Two types of positions of the impurity atom are considered. One is just below an edge of the ZGNR that is labeled the edge position, and the other is ~1.0 nm far inside the zigzag edge that is labeled the deep position.

## Supplementary information

Supplementary Information
