# Supplementary material for: Effects of intercalated atoms on electronic structure of graphene nanoribbon/hexagonal boron nitride stacked layer
Source: Sci Rep. 2019 Mar 6;9:3623. doi: 10.1038/s41598-019-39719-9 (PMC6403252; doi:10.1038/s41598-019-39719-9)
Supplement: Supplementary file 1 — Supplementary Information [file 41598_2019_39719_MOESM1_ESM.docx]

**Supplementary Information**

**Effect of intercalation atoms on electronic structure of graphene nanoribbon/hexagonal boron nitride stacked layer**

**Dongchul Sung**^1^**, Gunn Kim**^1,#^**,** **and Suklyun Hong**^1,*^

^1^ Department of Physics and Graphene Research Institute, Sejong University, Seoul 143-747, Korea

Corresponding author: [**^*^**hong@sejong.ac.kr](mailto:*hong@sejong.ac.kr), ^#^gunnkim@sejong.ac.kr


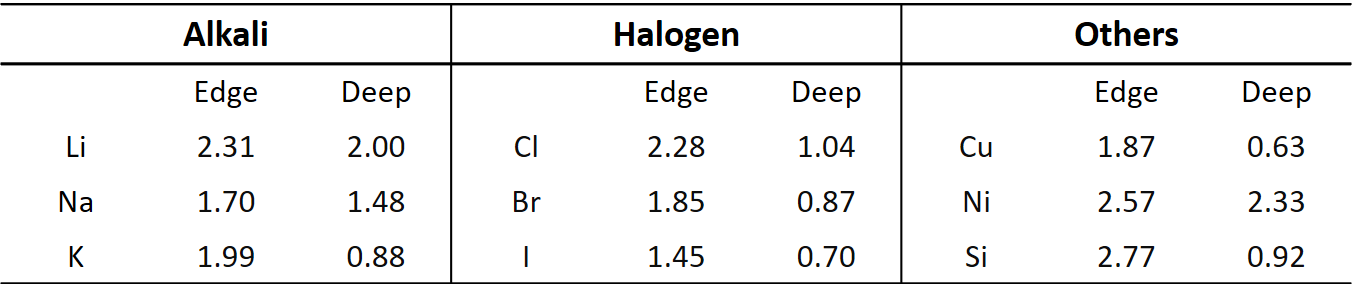


**Table S1.** Calculated binding energies (in eV) of the adsorbed atoms on the top surface of ZGNR in vdW ZGNR/h-BN heterostructures


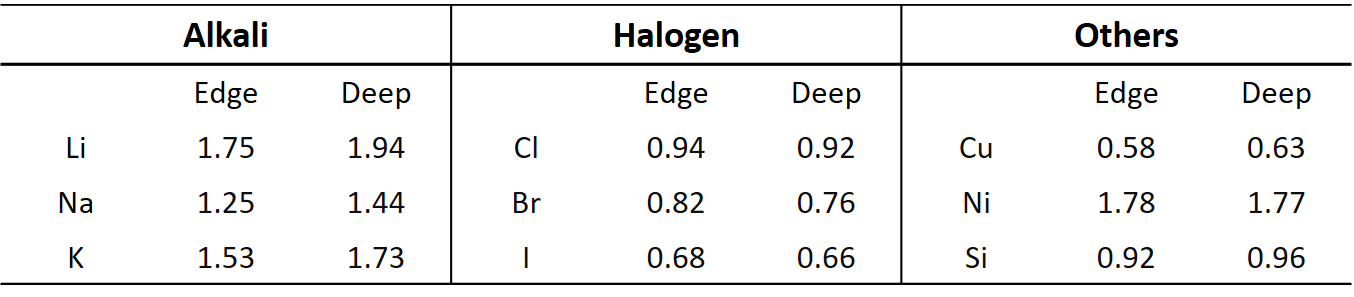


**Table S2.** Calculated binding energies (in eV) of the adsorbed atoms on the bottom surface of h-BN sheet in vdW ZGNR/h-BN heterostructures
